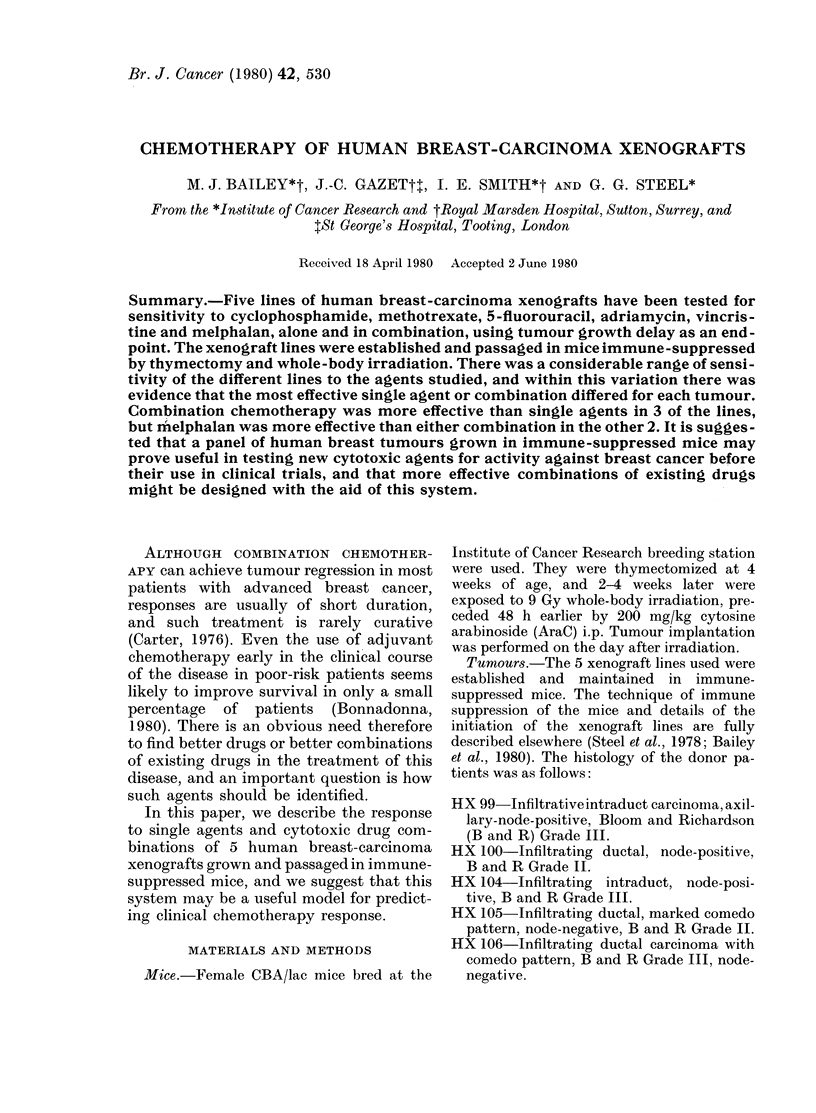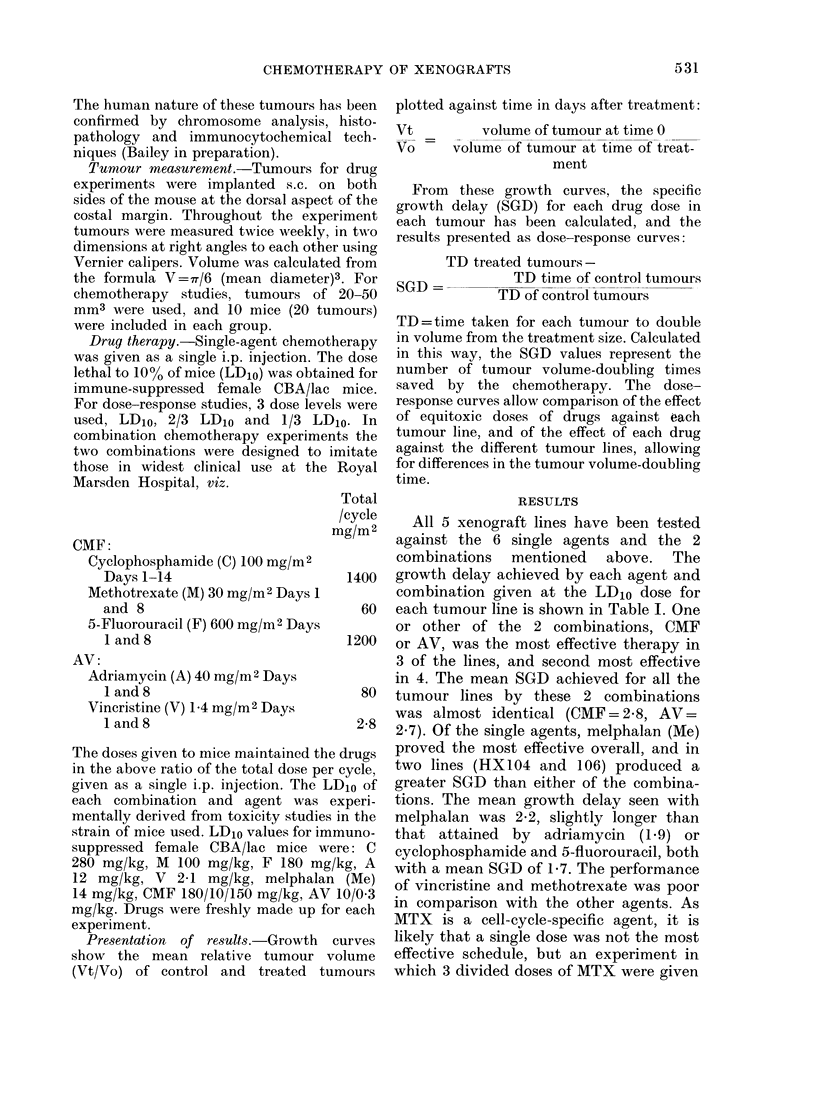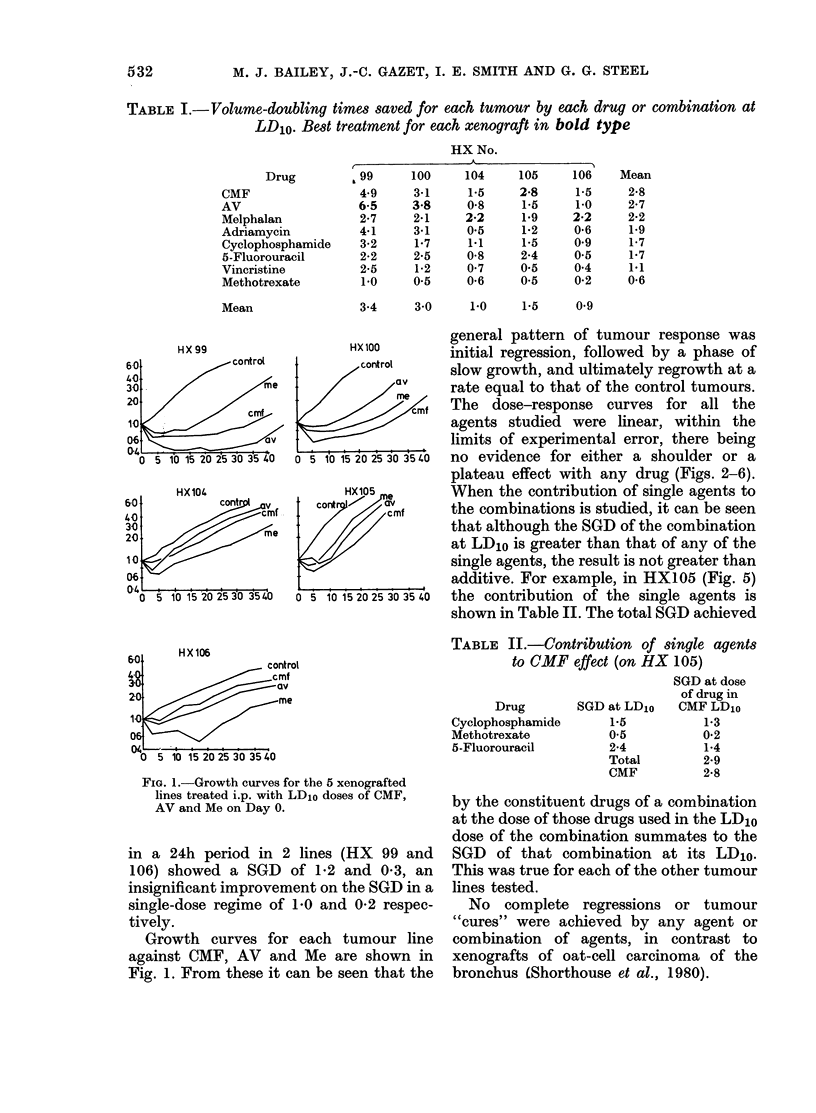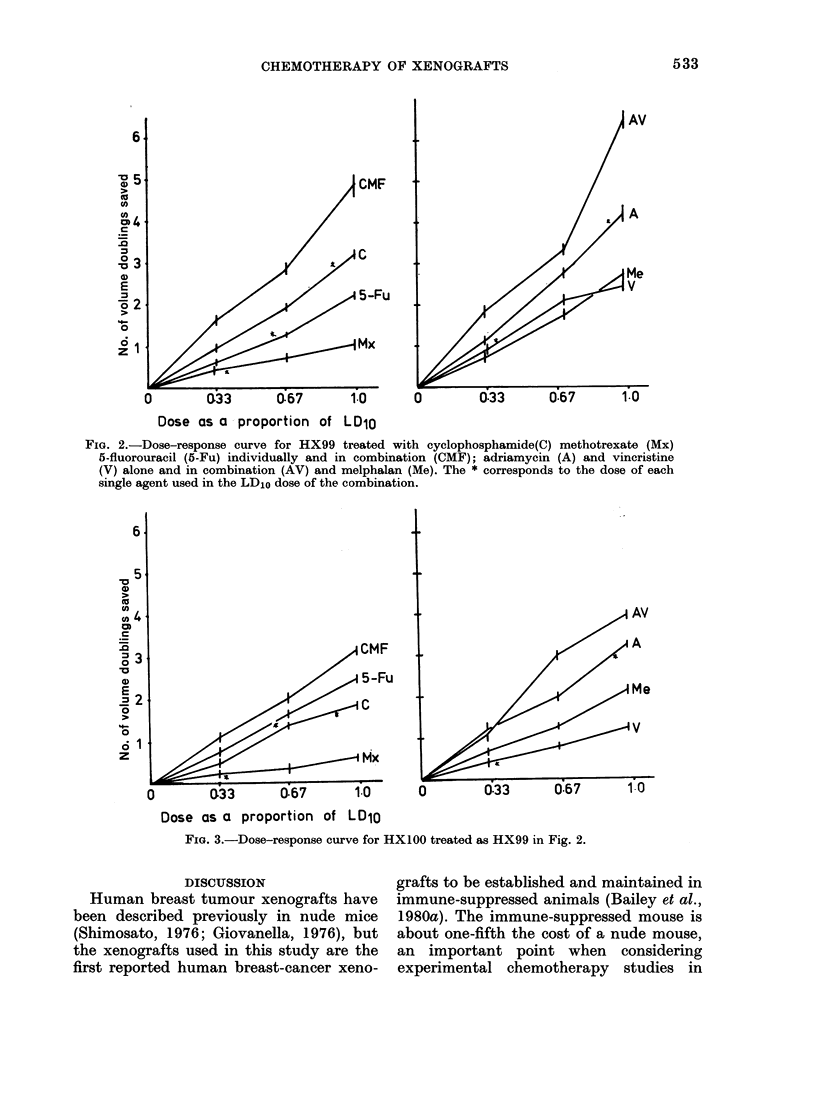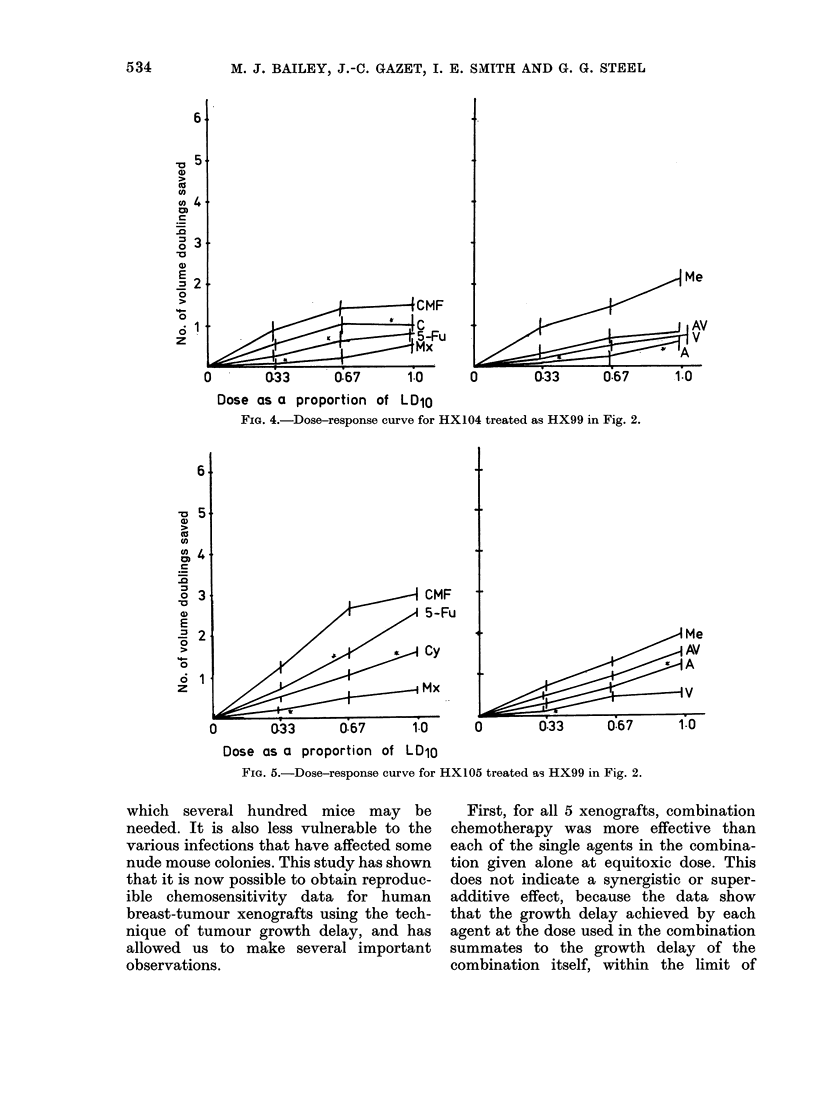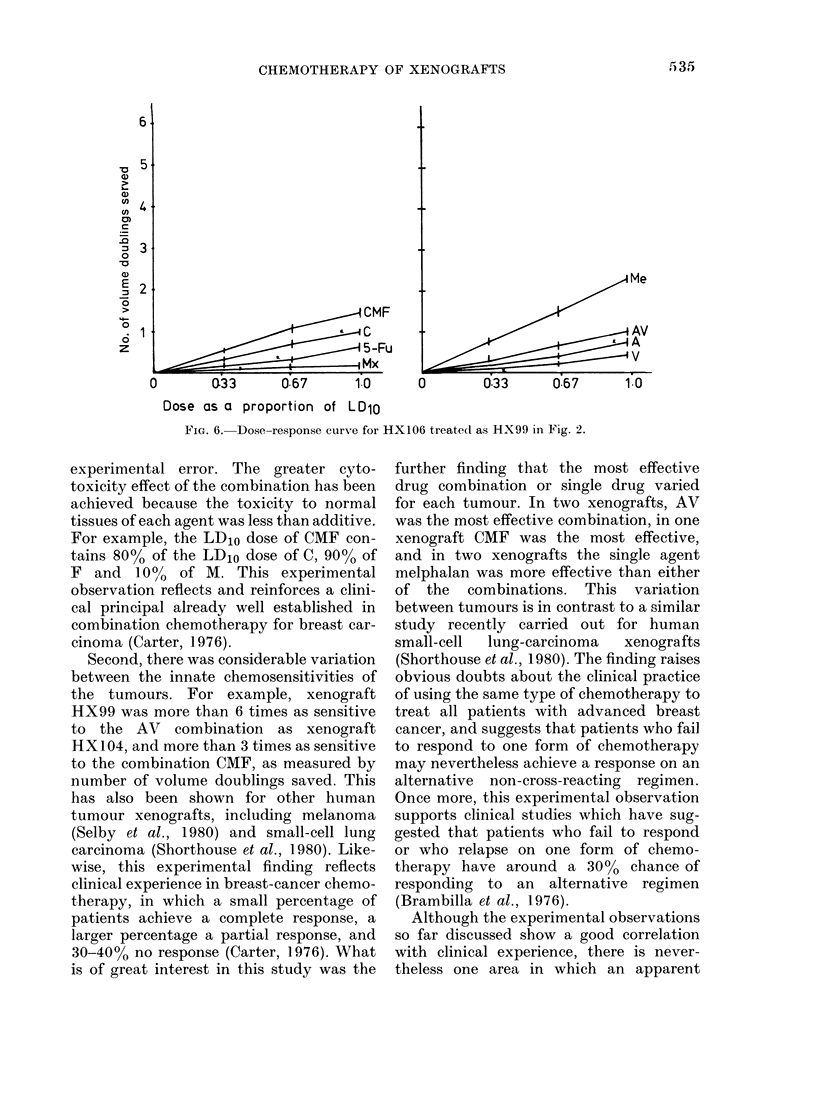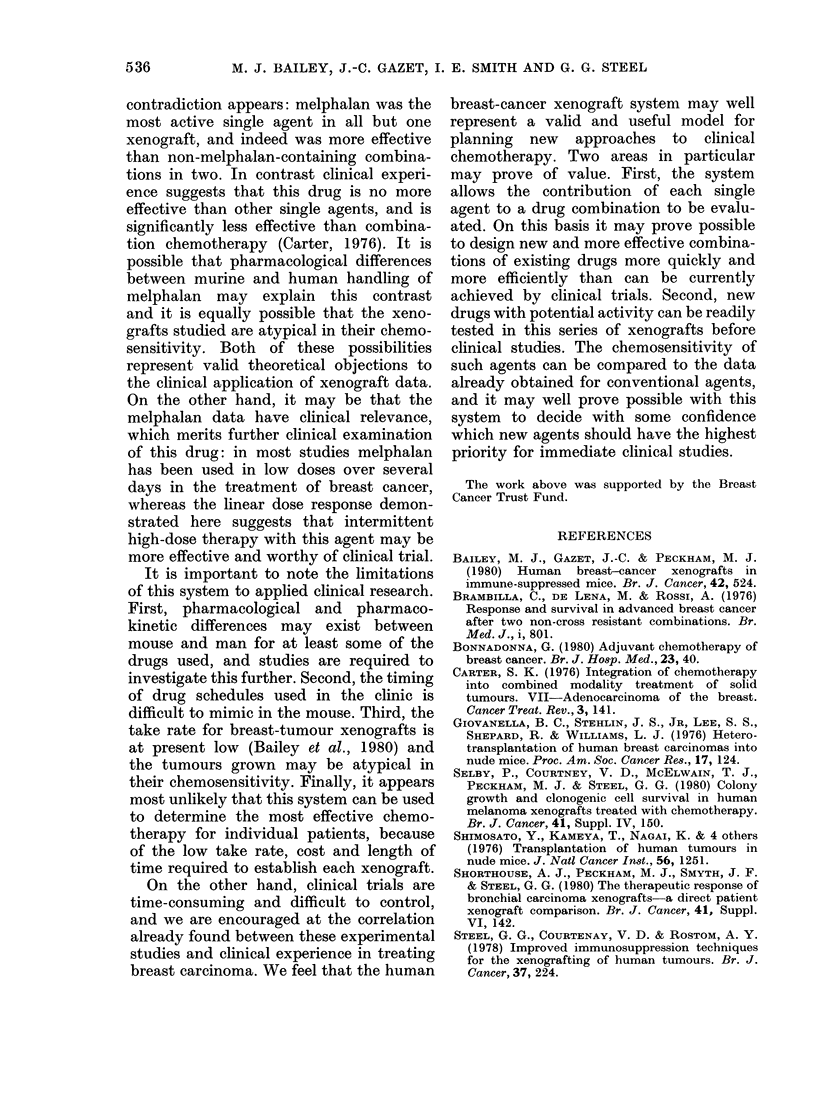# Chemotherapy of human breast-carcinoma xenografts.

**DOI:** 10.1038/bjc.1980.276

**Published:** 1980-10

**Authors:** M. J. Bailey, J. C. Gazet, I. E. Smith, G. G. Steel

## Abstract

Five lines of human breast-carcinoma xenografts have been tested for sensitivity to cyclophosphamide, methotrexate, 5-fluorouracil, adriamycin, vincristine and melphalan, alone and in combination, using tumour growth delay as an end-point. The xenograft lines were established and passaged in mice immune-suppressed by thymectomy and whole-body irradiation. There was a considerable range of sensitivity of the different lines to the agents studied, and within this variation there was evidence that the most effective single agent or combination differed for each tumour. Combination chemotherapy was more effective than single agents in 3 of the lines, but melphalan was more effective than either combination in the other 2. It is suggested that a panel of human breast tumours grown in immune-suppressed mice may prove useful in testing new cytotoxic agents for activity against breast cancer before their use in clinical trials, and that more effective combinations of existing drugs might be designed with the aid of this system.


*Br. J. Cancer* (1980) **42**, 530

### CHEMOTHERAPY OF HUMAN BREAST-CARCINOMA XENOGRAFTS

M. J. BAILEY*†, J.-C. GAZET†‡, I. E. SMITH*† AND G. G. STEEL*

*From the *Institute of Cancer Research and †Royal Marsden Hospital, Sutton, Surrey, and ‡St George's Hospital, Tooting, London*

Received 18 April 1980 Accepted 2 June 1980

**Summary.—Five lines of human breast-carcinoma xenografts have been tested for sensitivity to cyclophosphamide, methotrexate, 5-fluorouracil, adriamycin, vincristine and melphalan, alone and in combination, using tumour growth delay as an end-point. The xenograft lines were established and passaged in mice immune-suppressed by thymectomy and whole-body irradiation. There was a considerable range of sensitivity of the different lines to the agents studied, and within this variation there was evidence that the most effective single agent or combination differed for each tumour. Combination chemotherapy was more effective than single agents in 3 of the lines, but melphalan was more effective than either combination in the other 2. It is suggested that a panel of human breast tumours grown in immune-suppressed mice may prove useful in testing new cytotoxic agents for activity against breast cancer before their use in clinical trials, and that more effective combinations of existing drugs might be designed with the aid of this system.**

ALTHOUGH COMBINATION CHEMOTHERAPY can achieve tumour regression in most patients with advanced breast cancer, responses are usually of short duration, and such treatment is rarely curative (Carter, 1976). Even the use of adjuvant chemotherapy early in the clinical course of the disease in poor-risk patients seems likely to improve survival in only a small percentage of patients (Bonnadonna, 1980). There is an obvious need therefore to find better drugs or better combinations of existing drugs in the treatment of this disease, and an important question is how such agents should be identified.

In this paper, we describe the response to single agents and cytotoxic drug combinations of 5 human breast-carcinoma xenografts grown and passaged in immune-suppressed mice, and we suggest that this system may be a useful model for predicting clinical chemotherapy response.

### MATERIALS AND METHODS

*Mice.*—Female CBA/lac mice bred at the Institute of Cancer Research breeding station were used. They were thymectomized at 4 weeks of age, and 2–4 weeks later were exposed to 9 Gy whole-body irradiation, preceded 48 h earlier by 200 mg/kg cytosine arabinoside (AraC) i.p. Tumour implantation was performed on the day after irradiation.

*Tumours.*—The 5 xenograft lines used were established and maintained in immune-suppressed mice. The technique of immune suppression of the mice and details of the initiation of the xenograft lines are fully described elsewhere (Steel *et al.*, 1978; Bailey *et al.*, 1980). The histology of the donor patients was as follows:

HX 99—Infiltrative intraduct carcinoma, axillary-node-positive, Bloom and Richardson (B and R) Grade III.
HX 100—Infiltrating ductal, node-positive, B and R Grade II.
HX 104—Infiltrating intraduct, node-positive, B and R Grade III.
HX 105—Infiltrating ductal, marked comedo pattern, node-negative, B and R Grade II.
HX 106—Infiltrating ductal carcinoma with comedo pattern, B and R Grade III, node-negative.

The human nature of these tumours has been confirmed by chromosome analysis, histopathology and immunocytochemical techniques (Bailey in preparation).

*Tumour measurement.*—Tumours for drug experiments were implanted s.c. on both sides of the mouse at the dorsal aspect of the costal margin. Throughout the experiment tumours were measured twice weekly, in two dimensions at right angles to each other using Vernier calipers. Volume was calculated from the formula $V = \pi/6$ (mean diameter)$^3$. For chemotherapy studies, tumours of 20–50 $mm^3$ were used, and 10 mice (20 tumours) were included in each group.

*Drug therapy.*—Single-agent chemotherapy was given as a single i.p. injection. The dose lethal to 10% of mice ($LD_{10}$) was obtained for immune-suppressed female CBA/lac mice. For dose–response studies, 3 dose levels were used, $LD_{10}$, 2/3 $LD_{10}$ and 1/3 $LD_{10}$. In combination chemotherapy experiments the two combinations were designed to imitate those in widest clinical use at the Royal Marsden Hospital, *viz.*

| | Total /cycle mg/m$^2$ |
|---|---|
| CMF: | |
| Cyclophosphamide (C) 100 mg/m$^2$ Days 1–14 | 1400 |
| Methotrexate (M) 30 mg/m$^2$ Days 1 and 8 | 60 |
| 5-Fluorouracil (F) 600 mg/m$^2$ Days 1 and 8 | 1200 |
| AV: | |
| Adriamycin (A) 40 mg/m$^2$ Days 1 and 8 | 80 |
| Vincristine (V) 1·4 mg/m$^2$ Days 1 and 8 | 2·8 |

The doses given to mice maintained the drugs in the above ratio of the total dose per cycle, given as a single i.p. injection. The $LD_{10}$ of each combination and agent was experimentally derived from toxicity studies in the strain of mice used. $LD_{10}$ values for immunosuppressed female CBA/lac mice were: C 280 mg/kg, M 100 mg/kg, F 180 mg/kg, A 12 mg/kg, V 2·1 mg/kg, melphalan (Me) 14 mg/kg, CMF 180/10/150 mg/kg, AV 10/0·3 mg/kg. Drugs were freshly made up for each experiment.

*Presentation of results.*—Growth curves show the mean relative tumour volume (Vt/Vo) of control and treated tumours plotted against time in days after treatment:

$$\frac{Vt}{Vo} = \frac{\text{volume of tumour at time 0}}{\text{volume of tumour at time of treatment}}$$

From these growth curves, the specific growth delay (SGD) for each drug dose in each tumour has been calculated, and the results presented as dose–response curves:

$$SGD = \frac{\text{TD treated tumours} - \text{TD time of control tumours}}{\text{TD of control tumours}}$$

TD = time taken for each tumour to double in volume from the treatment size. Calculated in this way, the SGD values represent the number of tumour volume-doubling times saved by the chemotherapy. The dose–response curves allow comparison of the effect of equitoxic doses of drugs against each tumour line, and of the effect of each drug against the different tumour lines, allowing for differences in the tumour volume-doubling time.

### RESULTS

All 5 xenograft lines have been tested against the 6 single agents and the 2 combinations mentioned above. The growth delay achieved by each agent and combination given at the $LD_{10}$ dose for each tumour line is shown in Table I. One or other of the 2 combinations, CMF or AV, was the most effective therapy in 3 of the lines, and second most effective in 4. The mean SGD achieved for all the tumour lines by these 2 combinations was almost identical (CMF = 2·8, AV = 2·7). Of the single agents, melphalan (Me) proved the most effective overall, and in two lines (HX104 and 106) produced a greater SGD than either of the combinations. The mean growth delay seen with melphalan was 2·2, slightly longer than that attained by adriamycin (1·9) or cyclophosphamide and 5-fluorouracil, both with a mean SGD of 1·7. The performance of vincristine and methotrexate was poor in comparison with the other agents. As MTX is a cell-cycle-specific agent, it is likely that a single dose was not the most effective schedule, but an experiment in which 3 divided doses of MTX were given

TABLE I.—*Volume-doubling times saved for each tumour by each drug or combination at $LD_{10}$. Best treatment for each xenograft in* **bold type**

| Drug | HX No. 99 | 100 | 104 | 105 | 106 | Mean |
|---|---|---|---|---|---|---|
| CMF | 4·9 | 3·1 | 1·5 | **2·8** | 1·5 | 2·8 |
| AV | **6·5** | **3·8** | 0·8 | 1·5 | 1·0 | 2·7 |
| Melphalan | 2·7 | 2·1 | **2·2** | 1·9 | **2·2** | 2·2 |
| Adriamycin | 4·1 | 3·1 | 0·5 | 1·2 | 0·6 | 1·9 |
| Cyclophosphamide | 3·2 | 1·7 | 1·1 | 1·5 | 0·9 | 1·7 |
| 5-Fluorouracil | 2·2 | 2·5 | 0·8 | 2·4 | 0·5 | 1·7 |
| Vincristine | 2·5 | 1·2 | 0·7 | 0·5 | 0·4 | 1·1 |
| Methotrexate | 1·0 | 0·5 | 0·6 | 0·5 | 0·2 | 0·6 |
| Mean | 3·4 | 3·0 | 1·0 | 1·5 | 0·9 | |

FIG. 1.—Growth curves for the 5 xenografted lines treated i.p. with $LD_{10}$ doses of CMF, AV and Me on Day 0.

in a 24h period in 2 lines (HX 99 and 106) showed a SGD of 1·2 and 0·3, an insignificant improvement on the SGD in a single-dose regime of 1·0 and 0·2 respectively.

Growth curves for each tumour line against CMF, AV and Me are shown in Fig. 1. From these it can be seen that the general pattern of tumour response was initial regression, followed by a phase of slow growth, and ultimately regrowth at a rate equal to that of the control tumours. The dose–response curves for all the agents studied were linear, within the limits of experimental error, there being no evidence for either a shoulder or a plateau effect with any drug (Figs. 2–6). When the contribution of single agents to the combinations is studied, it can be seen that although the SGD of the combination at $LD_{10}$ is greater than that of any of the single agents, the result is not greater than additive. For example, in HX105 (Fig. 5) the contribution of the single agents is shown in Table II. The total SGD achieved

TABLE II.—*Contribution of single agents to CMF effect (on HX 105)*

| Drug | SGD at $LD_{10}$ | SGD at dose of drug in CMF $LD_{10}$ |
|---|---|---|
| Cyclophosphamide | 1·5 | 1·3 |
| Methotrexate | 0·5 | 0·2 |
| 5-Fluorouracil | 2·4 | 1·4 |
| | Total | 2·9 |
| | CMF | 2·8 |

by the constituent drugs of a combination at the dose of those drugs used in the $LD_{10}$ dose of the combination summates to the SGD of that combination at its $LD_{10}$. This was true for each of the other tumour lines tested.

No complete regressions or tumour "cures" were achieved by any agent or combination of agents, in contrast to xenografts of oat-cell carcinoma of the bronchus (Shorthouse *et al.*, 1980).

FIG. 2.—Dose–response curve for HX99 treated with cyclophosphamide(C) methotrexate (Mx) 5-fluorouracil (5-Fu) individually and in combination (CMF); adriamycin (A) and vincristine (V) alone and in combination (AV) and melphalan (Me). The * corresponds to the dose of each single agent used in the $LD_{10}$ dose of the combination.

FIG. 3.—Dose–response curve for HX100 treated as HX99 in Fig. 2.

### DISCUSSION

Human breast tumour xenografts have been described previously in nude mice (Shimosato, 1976; Giovanella, 1976), but the xenografts used in this study are the first reported human breast-cancer xenografts to be established and maintained in immune-suppressed animals (Bailey *et al.*, 1980*a*). The immune-suppressed mouse is about one-fifth the cost of a nude mouse, an important point when considering experimental chemotherapy studies in

FIG. 4.—Dose–response curve for HX104 treated as HX99 in Fig. 2.

FIG. 5.—Dose–response curve for HX105 treated as HX99 in Fig. 2.

which several hundred mice may be needed. It is also less vulnerable to the various infections that have affected some nude mouse colonies. This study has shown that it is now possible to obtain reproducible chemosensitivity data for human breast-tumour xenografts using the technique of tumour growth delay, and has allowed us to make several important observations.

First, for all 5 xenografts, combination chemotherapy was more effective than each of the single agents in the combination given alone at equitoxic dose. This does not indicate a synergistic or superadditive effect, because the data show that the growth delay achieved by each agent at the dose used in the combination summates to the growth delay of the combination itself, within the limit of

FIG. 6.—Dose-response curve for HX106 treated as HX99 in Fig. 2.

experimental error. The greater cytotoxicity effect of the combination has been achieved because the toxicity to normal tissues of each agent was less than additive. For example, the $LD_{10}$ dose of CMF contains 80% of the $LD_{10}$ dose of C, 90% of F and 10% of M. This experimental observation reflects and reinforces a clinical principal already well established in combination chemotherapy for breast carcinoma (Carter, 1976).

Second, there was considerable variation between the innate chemosensitivities of the tumours. For example, xenograft HX99 was more than 6 times as sensitive to the AV combination as xenograft HX104, and more than 3 times as sensitive to the combination CMF, as measured by number of volume doublings saved. This has also been shown for other human tumour xenografts, including melanoma (Selby *et al.*, 1980) and small-cell lung carcinoma (Shorthouse *et al.*, 1980). Likewise, this experimental finding reflects clinical experience in breast-cancer chemotherapy, in which a small percentage of patients achieve a complete response, a larger percentage a partial response, and 30–40% no response (Carter, 1976). What is of great interest in this study was the further finding that the most effective drug combination or single drug varied for each tumour. In two xenografts, AV was the most effective combination, in one xenograft CMF was the most effective, and in two xenografts the single agent melphalan was more effective than either of the combinations. This variation between tumours is in contrast to a similar study recently carried out for human small-cell lung-carcinoma xenografts (Shorthouse *et al.*, 1980). The finding raises obvious doubts about the clinical practice of using the same type of chemotherapy to treat all patients with advanced breast cancer, and suggests that patients who fail to respond to one form of chemotherapy may nevertheless achieve a response on an alternative non-cross-reacting regimen. Once more, this experimental observation supports clinical studies which have suggested that patients who fail to respond or who relapse on one form of chemotherapy have around a 30% chance of responding to an alternative regimen (Brambilla *et al.*, 1976).

Although the experimental observations so far discussed show a good correlation with clinical experience, there is nevertheless one area in which an apparent contradiction appears: melphalan was the most active single agent in all but one xenograft, and indeed was more effective than non-melphalan-containing combinations in two. In contrast clinical experience suggests that this drug is no more effective than other single agents, and is significantly less effective than combination chemotherapy (Carter, 1976). It is possible that pharmacological differences between murine and human handling of melphalan may explain this contrast and it is equally possible that the xenografts studied are atypical in their chemosensitivity. Both of these possibilities represent valid theoretical objections to the clinical application of xenograft data. On the other hand, it may be that the melphalan data have clinical relevance, which merits further clinical examination of this drug: in most studies melphalan has been used in low doses over several days in the treatment of breast cancer, whereas the linear dose response demonstrated here suggests that intermittent high-dose therapy with this agent may be more effective and worthy of clinical trial.

It is important to note the limitations of this system to applied clinical research. First, pharmacological and pharmacokinetic differences may exist between mouse and man for at least some of the drugs used, and studies are required to investigate this further. Second, the timing of drug schedules used in the clinic is difficult to mimic in the mouse. Third, the take rate for breast-tumour xenografts is at present low (Bailey *et al.*, 1980) and the tumours grown may be atypical in their chemosensitivity. Finally, it appears most unlikely that this system can be used to determine the most effective chemotherapy for individual patients, because of the low take rate, cost and length of time required to establish each xenograft.

On the other hand, clinical trials are time-consuming and difficult to control, and we are encouraged at the correlation already found between these experimental studies and clinical experience in treating breast carcinoma. We feel that the human breast-cancer xenograft system may well represent a valid and useful model for planning new approaches to clinical chemotherapy. Two areas in particular may prove of value. First, the system allows the contribution of each single agent to a drug combination to be evaluated. On this basis it may prove possible to design new and more effective combinations of existing drugs more quickly and more efficiently than can be currently achieved by clinical trials. Second, new drugs with potential activity can be readily tested in this series of xenografts before clinical studies. The chemosensitivity of such agents can be compared to the data already obtained for conventional agents, and it may well prove possible with this system to decide with some confidence which new agents should have the highest priority for immediate clinical studies.

The work above was supported by the Breast Cancer Trust Fund.

### REFERENCES

BAILEY, M. J., GAZET, J.-C. & PECKHAM, M. J. (1980) Human breast–cancer xenografts in immune-suppressed mice. *Br. J. Cancer*, **42**, 524.

BRAMBILLA, C., DE LENA, M. & ROSSI, A. (1976) Response and survival in advanced breast cancer after two non-cross resistant combinations. *Br. Med. J.*, i, 801.

BONNADONNA, G. (1980) Adjuvant chemotherapy of breast cancer. *Br. J. Hosp. Med.*, **23**, 40.

CARTER, S. K. (1976) Integration of chemotherapy into combined modality treatment of solid tumours. VII—Adenocarcinoma of the breast. *Cancer Treat. Rev.*, **3**, 141.

GIOVANELLA, B. C., STEHLIN, J. S., JR, LEE, S. S., SHEPARD, R. & WILLIAMS, L. J. (1976) Heterotransplantation of human breast carcinomas into nude mice. *Proc. Am. Soc. Cancer Res.*, **17**, 124.

SELBY, P., COURTNEY, V. D., MCELWAIN, T. J., PECKHAM, M. J. & STEEL, G. G. (1980) Colony growth and clonogenic cell survival in human melanoma xenografts treated with chemotherapy. *Br. J. Cancer*, **41**, Suppl. IV, 150.

SHIMOSATO, Y., KAMEYA, T., NAGAI, K. & 4 others (1976) Transplantation of human tumours in nude mice. *J. Natl Cancer Inst.*, **56**, 1251.

SHORTHOUSE, A. J., PECKHAM, M. J., SMYTH, J. F. & STEEL, G. G. (1980) The therapeutic response of bronchial carcinoma xenografts—a direct patient xenograft comparison. *Br. J. Cancer*, **41**, Suppl. VI, 142.

STEEL, G. G., COURTENAY, V. D. & ROSTOM, A. Y. (1978) Improved immunosuppression techniques for the xenografting of human tumours. *Br. J. Cancer*, **37**, 224.